# Sublingual Vaccination with Live Influenza Virus Induces Better Protection Than Oral Immunization in Mice

**DOI:** 10.3390/life12070975

**Published:** 2022-06-29

**Authors:** Jie Mao, Gi-Deok Eom, Keon-Woong Yoon, Hae-Ji Kang, Ki-Back Chu, Fu-Shi Quan

**Affiliations:** 1 Department of Biomedical Science, Graduate School, Kyung Hee University, Seoul 02447, Korea; maojie@khu.ac.kr (J.M.); ekd3910@naver.com (G.-D.E.); kgang92@gmail.com (K.-W.Y.); haedi1202@naver.com (H.-J.K.); 2 Medical Research Center for Bioreaction to Reactive Oxygen Species and Biomedical Science Institute, School of Medicine, Graduate School, Kyung Hee University, Seoul 02447, Korea; ckb421@gmail.com; 3 Department of Medical Zoology, School of Medicine, Kyung Hee University, Seoul 02447, Korea

**Keywords:** influenza virus, oral administration, sublingual administration, protection

## Abstract

Both sublingual (SL) and oral vaccine administration modalities are convenient, easy, and safe. Here, we have investigated the differences in vaccine efficacy that are induced by oral and sublingual immunization with live influenza virus (A/Hong Kong/1/1968, H3N2) in mice. Intranasally administering a lethal dose of the influenza virus resulted in the deaths of the mice, whereas viral replication in the lungs did not occur upon SL or oral administration. At 30 days post-immunization through the SL or oral route, the mice were intranasally challenge-infected with the lethal dose of the homologous influenza virus. Both SL and oral immunizations with the influenza virus elicited significantly higher levels of virus-specific IgG and IgA antibody responses, as well as HAI titers in the sera. Upon challenge infection, the SL immunization elicited higher levels of pulmonary IgG antibody and CD8^+^ T cell responses than the oral immunization. Enhanced splenic germinal center B (GC B) and B cell proliferation were also detected from the SL immunization, both of which were significantly greater than those of the oral immunization. Importantly, compared to oral immunization, significantly lessened lung viral loads and bodyweight reductions were observed from the SL immunization and these parameters contributed to prolonging the survival of the immunized mice. These results indicate that both SL and oral administration could be effective routes in inducing protective immunity against influenza virus infection, with SL immunization being the better of the two delivery routes.

## 1. Introduction

Seasonal influenza virus infections lead to approximately 3–5 million severe cases and 290,000–650,000 deaths on an annual basis throughout the globe [1,2]. Vaccination is the most effective way to prevent infection and the severe outcomes that are caused by influenza viruses. The World Health Organization (WHO) has recommended the administration of licensed influenza vaccines through intramuscular (IM) and intranasal (IN) routes, with the former of the two methods being more commonly utilized [3,4]. Although the IM immunization route is perceived to be effective, there are several disadvantages associated with it, which include pain at the injection site, a lack of mucosal immunity induction, a requirement of the presence of medical personnel, and others. IN immunization was also reported to cause mild influenza-like symptoms or even serious side effects, such as Bell’s palsy [4] or a redirection of antigens to the central nervous system [5]. Therefore, alternative vaccine delivery modalities that are safe, easy, and convenient for the patients, while maintaining high vaccine efficacy, are needed.

Mucosal vaccines, such as those that are delivered through the IN, sublingual (SL), and oral routes, are capable of inducing both systemic and mucosal antigen-specific immune responses [6]. This is one major advantage of mucosal vaccines over parental vaccines, which only elicit a systemic immune response in the immunized hosts [7]. IN delivery of influenza virus vaccines can induce potent mucosal immune responses and enhance protection [8], however, this immunization route is hindered by a short residence time, quick clearance of antigens, and can result in the retrograde passage of the vaccine components to the CNS [7]. Compared to IN, SL and oral routes are a safer, more convenient, and easier alternatives for vaccine administration.

The SL route is commonly used for immunotherapeutic treatments of allergies as it enables the rapid absorption of antigens that directly enter the bloodstream rather than bypassing through the gastrointestinal tract. While this approach can broadly disseminate immune responses, mass producing these types of vaccines is time consuming and technically challenging [9,10]. Importantly, it has been reported that SL immunization with live influenza virus-induced protection and was neither pathogenic nor incurred the migration of viruses to the CNS [9]. SL immunization is a safe and attractive option for mucosal vaccines. Oral vaccines can evoke immune responses in the small intestines, proximal colon, and salivary glands [6]. The orally-administered recombinant baculovirus-based influenza vaccine has been shown to induce mucosal and systemic immunity, thereby protecting the mice from the lethal influenza virus challenge infection [11]. Delivering a high dose of inactivated influenza vaccine through the oral route was reported to induce protection in mice [12]. Thus, we have hypothesized that the harsh environmental conditions of the gastrointestinal tract triggered the degradation of the inactivated influenza vaccine antigens and rendered them less immunogenic, while the live virus vaccines maintained their immunogenicity.

Although SL and oral administration routes are perceived to be the best in terms of safety and convenience, a direct comparative study evaluating the protective efficacy of the two methods using a live influenza virus vaccine is lacking. In this study, mice were orally or sublingually infected with a lethal dose of live influenza virus in order to confirm the absence of viral replication in the lungs. After that, the vaccinated mice were intranasally challenge-infected with a lethal dose of the influenza virus and the protective immunity was compared. Both SL and oral immunizations could be effective at inducing protection, however, the SL vaccination induces better protection than the oral administration.

## 2. Materials and Methods

### 2.1. Ethics Statement

Animal experiments in this study were carried out following the guidelines set out by Kyung Hee University IACUC (permit number: KHSASP-20-604). All efforts have been made to lower the number of animals used and to reduce their suffering.

### 2.2. Cells and Viruses

Madin–Darby canine kidney (MDCK) cells were grown and maintained in Dulbecco’s modified Eagle’s medium (DMEM; Welgene, Daegu, Korea) supplemented with 10% fetal bovine serum and 1% penicillin/streptomycin at 37 °C with 5% CO_2_. Human influenza A/Hong Kong/1/1968 (H3N2) virus was propagated as described [13]. Briefly, viruses were propagated in the allantoic cavity of 11-day-old embryonated chicken eggs. At 4 days post-infection (dpi), allantoic fluid containing the virus was harvested and purified using the 60%/30%/15% sucrose density gradient. The viruses were titrated, aliquoted, and stored at −80 °C until use. Formalin-inactivated viruses were used as coating antigens in the enzyme-linked immunosorbent assay (ELISA) and as a stimulant for flow cytometry, as previously described [1].

### 2.3. Primary Administration of the Virus into Mice and Challenge Infection

A total of 128 seven-week-old female BALB/c mice were purchased from NARA Biotech (Seoul, Korea). Of the 128 mice, 48 mice were used to confirm the safety of the oral and SL immunization methods in comparison to IN immunization at 100 PFU and 500 PFU infection doses (n = 8 per group). Of the 8 mice in each group, 4 mice were sacrificed to evaluate lung virus titer while the remaining 4 were used to measure changes in bodyweight and survival. The remaining 80 mice were evenly subdivided into 5 groups as follows (n = 16 per group): oral immunization (Oral), sublingual immunization (SL), intranasal immunization (IN) (which served as positive controls), an unimmunized infection control group (Naïve + Cha), and an unimmunized control group (Naive). All of the immunization groups were initially immunized with 100 PFU of live influenza (A/Hong Kong/1/1968 (H3N2)). At 30 days post-immunization, 8 mice from each immunization group were homologously challenge-infected with 200 PFU (2LD_50_), while the remaining 8 mice were infected with 1000 PFU (10LD_50_). Four mice from each group were randomly selected and sacrificed at 4 dpi for sample collection, while the remaining mice were monitored to assess bodyweight changes and survival rates until 14 dpi. The humane intervention point was defined as bodyweight loss exceeding 25% of its initial value and, upon reaching this endpoint, the mice were humanely euthanized with CO_2_ instillation.

### 2.4. Sample Collection and Preparation

Blood samples were obtained by retro-orbital plexus puncture at 3 weeks post-immunization. Sera were acquired after centrifuging the blood samples at 6000 rpm for 10 min at 4 °C, which were subsequently stored at −20 °C until use. Spleens and lungs were collected from 4 randomly selected mice at 4 dpi. Tissues were homogenized and dissociated cell suspensions were passed through 100 μm cell strainers. Lung homogenates were centrifuged at 4 °C at 3000 rpm for 10 min. Supernatants were carefully collected and stored at −80 °C to determine the viral titer and lung cytokine profiles, as previously described [11]. Pelleted lung cells were purified using 44–67% Percoll gradient (Cytiva, Marlborough, MA, USA) and were used for flow cytometry [14]. Dissociated splenocytes were resuspended in RBC lysis buffer (Sigma-Aldrich, St. Louis, MO, USA) and subsequently used for flow cytometric analysis.

### 2.5. Lung Viral Titer and Inflammatory Cytokine Responses

Lung virus titer and inflammatory cytokine responses were determined from homogenized lung extracts collected at 4 dpi through plaque assay or ELISA, as previously described [15]. Briefly, confluent monolayers of MDCK cells seeded in 6-well plates were incubated with serially-diluted lung supernatant samples at room temperature for 1 h. After aspirating the lung supernatants, the cells were gently overlaid with 1 mL of overlay media and incubated at 37 °C with 5% CO_2_ for 4 days. Once the overlay media had been removed, cells were fixed with 0.25% glutaraldehyde, and plaques were counted after staining with 1% crystal violet solution. Inflammatory cytokines interferon-gamma (IFN-γ) and interleukin-6 (IL-6) were measured following the manufacturer’s instructions provided in the BD OptEIA ELISA kits (BD Biosciences, San Jose, CA, USA).

### 2.6. Antibody Responses and Hemagglutination Inhibition (HAI) Titer

Influenza A/Hong Kong/1/1968 virus-specific IgG and IgA responses and HAI titer were measured by ELISA, as previously reported [15]. In brief, inactivated A/Hong Kong/1/1968 virus was resuspended in carbonate coating buffer and used to coat 96-well microtiter plates (SPL Life Sciences, Pocheon, Korea) at a concentration of 4 μg/mL, overnight at 4 °C. Wells were blocked with 0.2% gelatin dissolved in phosphate-buffered saline with 0.05% Tween-20 (PBST) and then incubated with either sera (1:50 dilution in PBS) or lung homogenates (1:10 dilution in PBS). After incubating the plates for 1 h at 37 °C, wells were washed and incubated with horseradish peroxidase (HRP)-conjugated anti-mouse secondary antibodies (1:2000 dilution in PBS; Southern Biotech, Birmingham, AL, USA) for 1 h at 37 °C. O-phenylenediamine substrate was dissolved in citrate-phosphate buffer with H_2_O_2_ and 100 μL of this solution was added to each well. Absorbance readings at 450 nm were measured using an EZ Read 400 microplate reader (Biochrom Ltd., Cambridge, UK). For HAI titers, sera were mixed with the receptor-destroying enzyme (Denka Seiken, Tokyo, Japan) at a ratio of 1:3 overnight at 37 °C and the enzyme was inactivated by heating at 56 °C for 30 min. Serum samples were then 2-fold serially diluted in a round-bottom 96-well plate and incubated at room temperature for 30 min after mixing with 4 HA units (HAU) of viruses. To each well, 0.5% chicken red blood cells (cRBCs) were added, and HAI titer was determined. HAI titers were defined as the reciprocal value of the highest serum dilution preventing hemagglutination.

### 2.7. Flow Cytometry

Flow cytometry was performed following the method previously described [14]. Splenocytes and lung cells acquired from the mice were individually processed to assess changes in immune cell populations. Cells were stimulated with 5 μg/mL of inactivated influenza virus for 2 h at 37 °C in complete RPMI media. Afterward, cells were washed and resuspended in stain buffer containing 0.2% bovine serum albumin (BSA) and 0.01% sodium azide in PBS for Fc-receptor blocking. Cells were incubated at 4 °C for 30 min with CD3-Cy5, CD4-FITC, CD8-PE, GL7-PE, B220-FITC, CD19-Cy7, and IgD-PE antibodies, which were purchased from BD Biosciences (San Jose, CA, USA). Afterward, cells were washed and fixed using 4% paraformaldehyde. Cells were acquired using the Accuri C6 flow cytometer and populations were analyzed by C6 Analysis Software (BD Biosciences, San Jose, CA, USA).

### 2.8. Statistical Analysis

Statistical analysis was performed using the Prism 5 software (GraphPad Software, Inc., San Diego, CA, USA). Data sets were presented as mean ± SD and a one-way analysis of variance (ANOVA) with Bonferroni’s post hoc test was used to determine the statistical significance between the groups. Asterisks were used to denote statistical significance and *p* values of less than 0.05 were considered statistically significant (* *p* < 0.05, ** *p* < 0.01, and *** *p* < 0.001).

## 3. Results

### 3.1. Lethal Dose of Influenza Virus Administration by SL or Oral Route Showed No Virus Replication and No Bodyweight Loss

**Figure 1:**
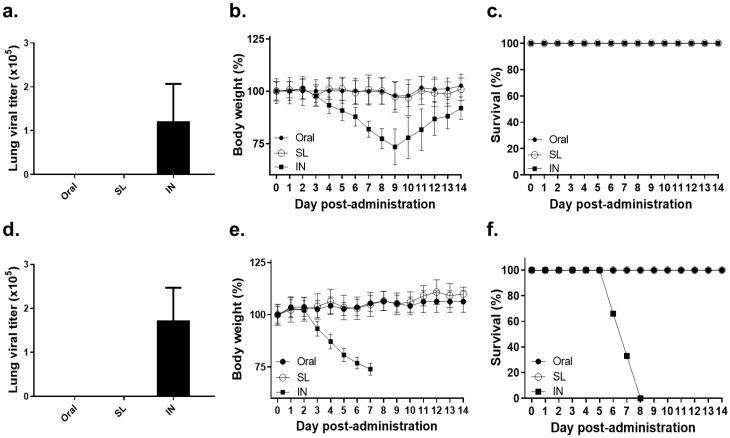
Oral or sublingual administration with a lethal dose of live influenza virus were safe. Mice (n = 8 per group) were immunized with live influenza virus (A/Hong Kong/1/1968 (H3N2) with either 100 PFU or 500 PFU by oral, SL, or IN routes. From each group, 4 mice were sacrificed 4 days after virus inoculation and lung virus titers were determined (**a**,**d**). The remaining 4 mice were monitored for 14 days to record changes in bodyweight (**b**,**e**) and survival (**c**,**f**). Data are expressed as mean ± SD from four independent experiments.

The mice were inoculated with either low (100 PFU) or high (500 PFU) doses of live influenza viruses through SL or oral routes in order to confirm whether viral infection through these modalities is possible. At four dpi, lung tissues were collected and viral titer, as well as bodyweight changes, were compared to the IN positive control mice. Neither the SL nor the oral administration of the low dose of live influenza virus resulted in viral replication in the lungs, whereas lung virus titers were detected in the IN positive control group (Figure 1a). Consistent with this finding, the bodyweight changes were negligible in the SL and oral groups, whereas a gradual bodyweight reduction was observed in the IN positive control group over the first few days (Figure 1b). However, the IN mice eventually regained their bodyweight and none of the mice in all three of the groups perished (Figure 1c). Similar to the low dose of infection, the high dose of infection through the SL or oral routes did not elicit lung viremia. The viral titer elicited by a high dose of influenza virus inoculation through the IN route was comparable to that of the low dose of infection (Figure 1d). However, intranasally administering a high dose of influenza virus resulted in the deaths of mice by eight dpi, while all of the mice belonging to the SL and oral groups survived (Figure 1e,f). These results have demonstrated that oral or SL virus exposure does not incur viral replication in the lungs, regardless of infection dose, thereby confirming that these administration routes are safe.

### 3.2. SL or Oral Administrations Elicited Virus-Specific Antibody Responses and HAI Titer

**Figure 2:**
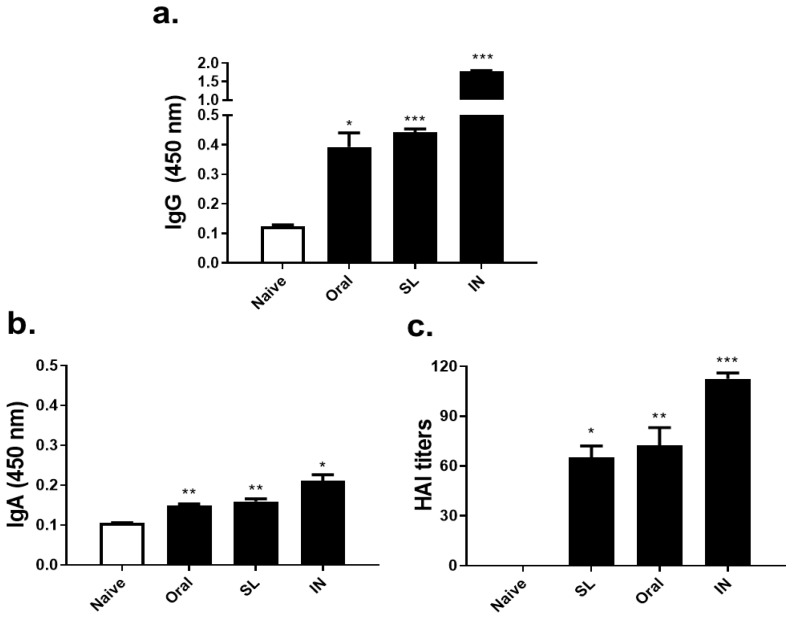
Antibody responses in sera. Virus-specific IgG (**a**), IgA (**b**), and hemagglutinin-inhibition titers (**c**) were determined in sera collected at day 21 post-immunization with the live virus (1000 PFU/mouse) by SL, oral, or IN routes. Data are expressed as mean ± SD (* *p* < 0.05, ** *p* < 0.01, *** *p* < 0.001 compared to naïve control) and are representative of four independent experiments.

At three weeks after live virus administration, sera were collected in order to determine virus-specific antibody responses and HAI titer. Virus-specific IgG antibody responses elicited upon SL or oral live virus immunization were significantly greater than that of the unimmunized control group (Figure 2a). SL and oral immunization also induced virus-specific IgA antibody responses, albeit being induced to a much lower extent than the IgG levels (Figure 2b). As expected, HAI titers were not detected from the naïve control, however, the SL and oral immunization induced HAI titers ranging between 60 and 80 (Figure 2c). While both oral and SL immunization induced antibody responses and HAI titers, significant differences between the two groups were not detected. Compared to these two administration routes, IN immunization as the positive control demonstrated substantially higher antibody responses and HAI titers.

### 3.3. SL and Oral Virus Inoculation Induced Virus-Specific Antibody Responses in the Lungs

**Figure 3:**
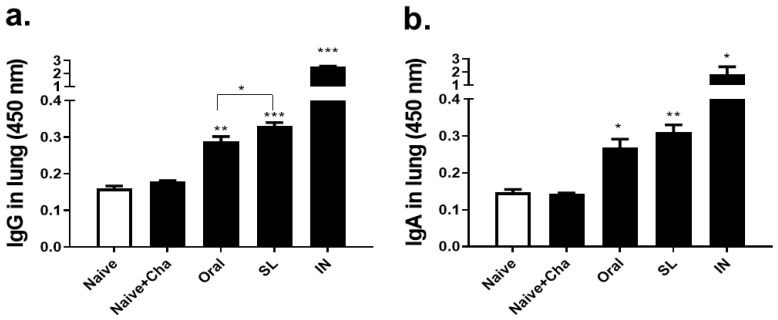
Virus-specific antibodies in the lungs of mice. Immunized mice were challenge-infected with influenza viruses (1000 PFU/mouse) and lungs were collected at 4 dpi to measure virus-specific IgG (**a**) and IgA (**b**) antibody responses in the lungs. Data are expressed as mean ± SD (* *p* < 0.05, ** *p* < 0.01, *** *p* < 0.001 compared to Naïve + Cha) and are representative of four independent experiments.

At four dpi, lung supernatants were collected from the mice that were challenge-infected with 10 LD_50_ of A/Hong Kong/1/1968 virus and lung antibody responses were determined by ELISA. The SL and oral immunization with the live virus contributed to enhancing the virus-specific IgG (Figure 3a) and IgA (Figure 3b) levels in the lungs of the mice. Both of the immunization routes elicited antibody responses that were significantly higher than those that were induced in Naïve and Naïve + Cha groups. Lung IgG responses evoked through the SL virus immunization were significantly greater than those of the oral immunization, but such findings were not detected for IgA.

### 3.4. SL and Oral Administrations Induced Cellular Immune Responses in the Lungs

**Figure 4:**
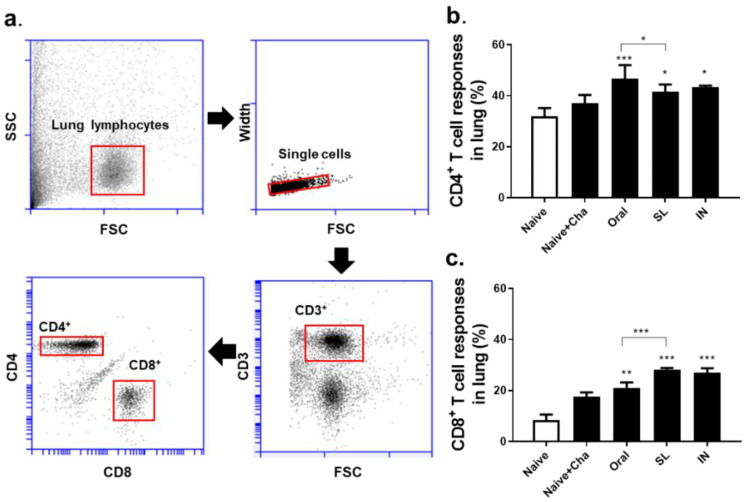
T cell responses in the lungs of mice. Single lung cell suspension collected at 4 dpi were strategically gated (**a**) to evaluate CD4^+^ and CD8^+^ T cell responses via flow cytometry (**b**,**c**). Representative data from four independent experiments are displayed as mean ± SD (* *p* < 0.05, ** *p* < 0.01, *** *p* < 0.001 compared to Naïve + Cha).

In order to measure the efficacy of SL and oral live virus immunization, mice were sacrificed at four dpi and lung cells were collected for flow cytometry analysis. Single cell populations of lung cells were gated in order to assess CD4^+^ and CD8^+^ T cell proliferation (Figure 4a). Compared to the unimmunized controls, oral and SL immunization enhanced CD4^+^ T cell proliferation with higher CD4^+^ T cell response being observed in the former of the two. The IN immunization-induced T cell responses were comparable to both the SL and the oral routes (Figure 4b). Similarly, all of the immunization routes enhanced the CD8^+^ T cell responses in the lungs. Notably, the SL immunization induced significantly higher CD8^+^ T cell responses than the oral or the unimmunized controls (Figure 4c).

### 3.5. SL and Oral Administrations Evoked GC B Cell and B Cell Responses in Lungs and Spleens

**Figure 5:**
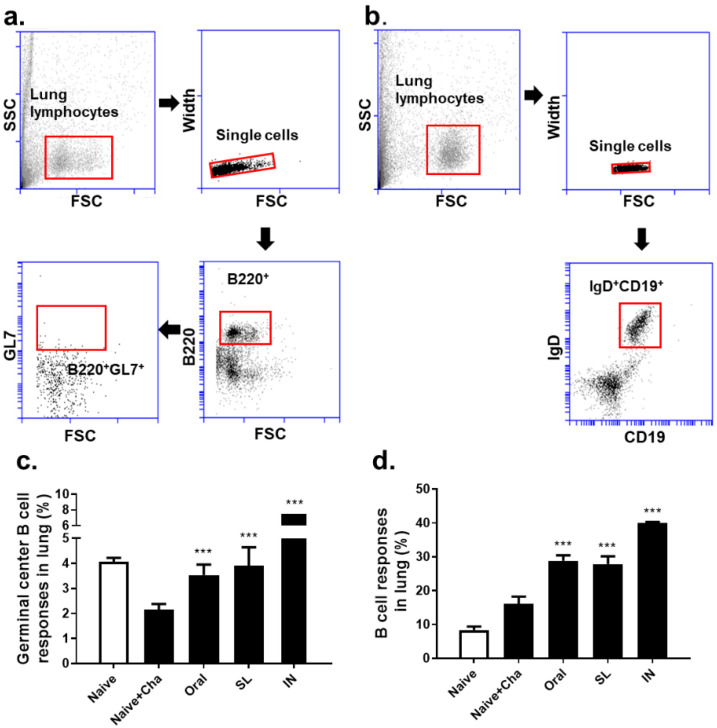
Germinal center B cell and B cell responses in the lungs of mice. Lung cells were collected at 4 dpi, and cells were gated (**a**,**b**). Populations of GC B cells (**c**) and B cells (**d**) were determined by flow cytometry. Representative data from four independent experiments are displayed as mean ± SD (*** *p* < 0.001 compared to Naïve + Cha).

**Figure 6:**
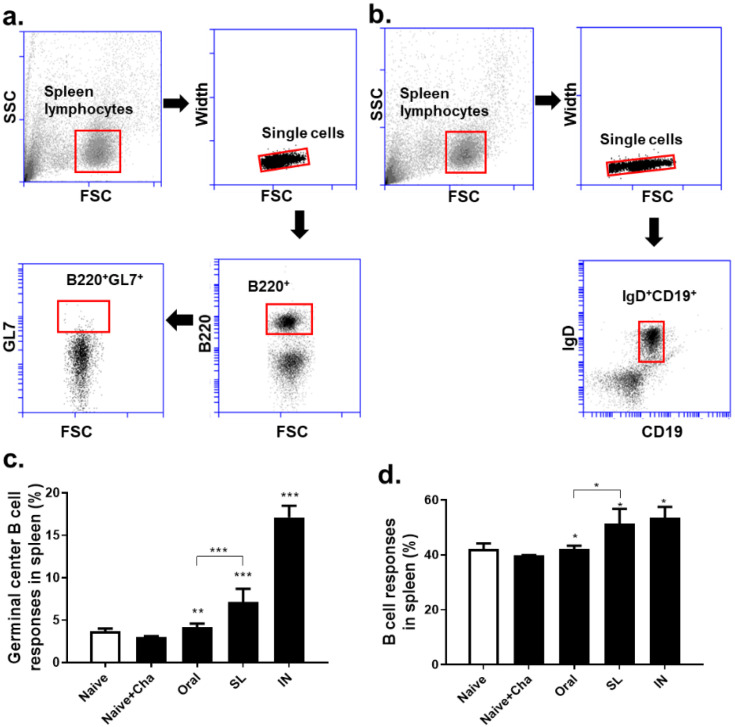
Germinal center B cell and B cell responses in the spleens of mice. Gating strategy to identify germinal center cells (**a**) and B cells (**b**) in spleens are shown. The percentages of germinal center B cells (**c**) and B cell populations (**d**) were determined by flow cytometry. Representative data from four independent experiments are expressed as mean ± SD (* *p* < 0.05, ** *p* < 0.01, *** *p* < 0.001 compared to Naïve + Cha).

Single cell populations of murine lung cells were gated in order to evaluate the changes in the GC B and B cells (Figure 5a,b). In the Naïve + Cha group, a marked reduction in the GC B cell population was observed. The oral and SL immunization prevented this phenomenon from occurring, as the GC B cell populations in these two groups were maintained at levels similar to that of the Naïve control (Figure 5c). Immunizing the mice with the live virus drastically enhanced the B cell responses in the lungs, irrespective of the administration route (Figure 5d). Splenocytes were also analyzed in order to assess the GC B and B cell populations using the identical gating strategy (Figure 6a,b). Similar to the lungs, a marginal decrease in the GC B cell population was observed in the Naïve + Cha group. Both the oral and the SL immunization increased the GC B cell proliferation in the splenocytes. A marked difference was noted between the oral and SL groups, with significantly higher GC B levels in the latter (Figure 6c). Likewise, homologous virus challenge infection resulted in a significantly higher B cell response in the SL route than in the oral immunization route (Figure 6d).

### 3.6. SL and Oral Administrations Lessened the Lung Inflammatory Responses

**Figure 7:**
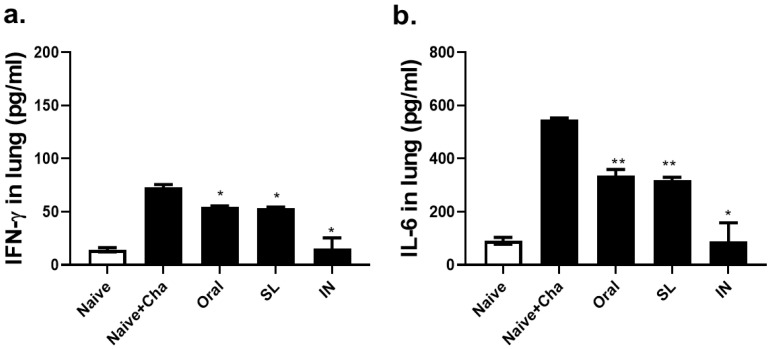
Pro-inflammatory cytokine responses in the lungs. Levels of interferon-gamma (**a**) and interleukin-6 (**b**) were detected in lung extracts at 4 dpi. Data are representative of four independent experiments and are expressed as mean ± SD (* *p* < 0.05, ** *p* < 0.01 compared to Naïve + Cha).

In order to confirm whether the oral and the SL immunization could suppress the inflammatory response incurred through viral infection, pro-inflammatory cytokines, interferon-gamma (IFN-γ) and interleukin-6 (IL-6), levels were assessed in the lung homogenates. Compared to Naïve + Cha control, both the oral and the SL immunization lessened the expression of the pulmonary IFN-γ levels to a significant extent (Figure 7a). A similar phenomenon was observed for IL-6, as the immunization with the live virus reduced the IL-6 expression, regardless of the administration route (Figure 7b). For both of the cytokines, a significant difference between the oral and the SL groups was not detected.

### 3.7. SL and Oral Immunization Diminished Lung Viral Load and Fully Protected Mice against a Low Dose of Infection but Conferred Partial Protection against a High Dose of Infection

**Figure 8:**
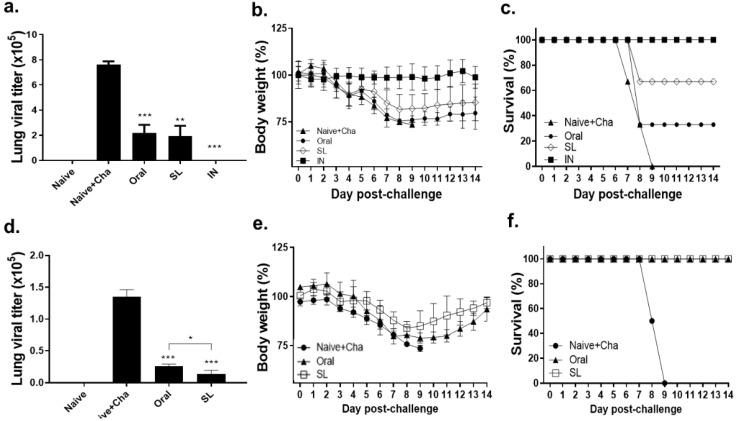
Lung viral titer and protective efficacy of live virus vaccine upon a high or low dose of challenge infection. Immunized mice were challenge-infected with either a high dose (1000 PFU, 10LD_50_) or a low dose (200 PFU, 2LD_50_). Lung viral burdens (**a**,**d**) were determined at 4 dpi. Bodyweight (**b**,**e**) and survival (**c**,**f**) of mice were monitored daily until 14 dpi. Data are displayed as mean ± SD (* *p* < 0.05, ** *p* < 0.01, *** *p* < 0.001 compared to Naïve + Cha) and are representative of four independent experiments.

One month after being immunized with 100 PFU of live A/Hong Kong/1/1968(H3N2) influenza virus, the mice were homologously challenge infected with a high (10LD_50_) or low (2LD_50_) dose of the virus. Upon the high dose of infection, the lung virus titers in the oral and the SL groups underwent an approximately 4-fold reduction in comparison to the Naïve + Cha group, while the viral plaques were undetected in the IN positive control group (Figure 8a). While the bodyweight changes were negligible in the IN group, the mice receiving either the oral or the SL immunization experienced substantial bodyweight loss from three dpi onward. The bodyweight reductions in the oral immunization group nearly approached the humane intervention point, while they were maintained at a slightly higher level in the SL immunization group. In both the oral and the SL groups, gradual bodyweight recovery began to occur from nine dpi onward, while all of the mice in the Naïve + Cha group perished by this time point (Figure 8b). None of the mice survived the high dose of challenge infection in the Naïve + Cha group. The survival rates for the oral and SL groups were 33% and 67%, respectively. None of the mice died when immunized through the IN route (Figure 8c). A drastic reduction in the viral titers was detected in both the oral- and the SL-immunized mice (Figure 8d). While substantial bodyweight loss was detected from both the oral and SL groups, this was followed by a recovery phase (Figure 8e). None of the immunized mice perished, while all of the Naïve + Cha mice died by nine dpi (Figure 8f). These results suggest that, upon a relatively low dose of infection, oral and SL immunization with the live virus can confer adequate protection, but it is insufficient to confer full protection against a high dose of infection.

## 4. Discussion

In the present study, we have confirmed that administering live A/Hong Kong/1/1968 (H3N2) influenza virus in mice through SL or oral routes can induce immune responses. This has contributed to conferring protection to an extent in mice upon challenge infection with the homologous virus.

The safety of vaccines, which largely depends on administration routes, is one of the factors that must be taken into consideration in the vaccine design. In our study, safety concerns were posed by neither the entry of the live influenza virus through the gastric tract nor via the diffusion under the tongue. However, several safety issues concerning IN immunization of influenza-related vaccines have been reported, including Bell’s palsy, which is caused by the retrograde passage of inhaled antigens into the brain [9,16], as well as the severe viral burden in the lungs in the case of live virus vaccines. In general, placing antigens under the tongue can enable their rapid uptake, which occurs within 15 to 20 min after contact, as the blood vessels are abundant in these areas. Antigens that are administered by the SL route are uptaken by the dendritic cells and other antigen-presenting cells within the sublingual epithelium and elicit their maturation. These cells migrate to the proximal draining lymph nodes, which enables the induction and dissemination of the antigen-specific host immune response from local to distant mucosal sites [17,18]. In addition, they are absorbed directly into the bloodstream upon contact with the oral mucosa in order to induce a systemic immune response [19]. Multiple studies have demonstrated that sublingual immunization can induce robust immune responses and effective protection against reinfection [8,9,20,21], which is in agreement with our study. However, perceptions of oral influenza vaccines among researchers are divided to a certain extent, owing to the challenges that are associated with antigenic degradation in the harsh gastrointestinal (GI) environment that can ultimately result in the loss of vaccine efficacy [16,22,23]. According to our results, the SL administration showed greater effectiveness in eliciting IgG and CD8^+^ T cell responses in the lungs than those that were induced by oral inoculation, which in part contributed to the higher protective efficacy of the SL immunization. Specific IgG antibodies in the lungs work more intensively on viruses, primarily attacking the lower respiratory epithelium, resulting in the loss of viral infectivity. Furthermore, these aid in restraining viral replication through antibody-dependent cell-mediated cytotoxicity (ADCC) within a shorter time and at a greater magnitude than serum antibodies, owing to the compartmentalization of the immune responses [2,24]. Similarly, CD8^+^ T cell responses act on local sites to limit the viral spread and to remove infection through the direct killing of virus-infected cells [25]. In addition, in our study, the SL administration induced a greater proportion of germinal center B cells and B cells in the spleens than the oral administration, which may also contribute to the better protection efficacy of the SL immunization than the oral vaccine administration. Previously, one study elucidated that the distinct dynamics and mechanisms of GC B cells depended on different organs, as well as total B cells [26]. It was suggested that GC responses in spleens peak faster than in other organs, which was in line with our results that were obtained on four dpi. Prior studies have also reported that the spleen harbored the largest proportion of GC B cells after the influenza virus infection, which contributes to delivering long-term protection against reinfection [27,28]. Based on our findings, in addition to the numerous reports documenting pulmonary immune responses induced by influenza vaccines [29,30,31], we supposed that the magnitude of GC B and B cells within spleens may be meaningful indicators mirroring protective efficacy. Whether or not additional immunization or adjuvant usage enhances immune responses and the protective efficacy of oral vaccines against a high dose of challenge infection remains a question to be addressed in further studies.

The FDA-approved quadrivalent nasal spray vaccine FluMist (MedImmuune) is based on live-attenuated influenza viruses and is commercially available in several countries [32]. Based on the data that is presented here, and also from another study [9], the SL immunization with the live influenza virus is considered to be highly effective and safe. Furthermore, unlike intranasal delivery with influenza vaccines, the risk of potential CNS damage is non-existent for SL immunization. To this extent, SL immunization with live or attenuated influenza virus possesses great potential and could signify viable application in practice.

## Data Availability

Not applicable.
